# Enhancing the thermostability of *Streptomyces cyaneofuscatus* strain Ms1 tyrosinase by multi-factors rational design and molecular dynamics simulations

**DOI:** 10.1371/journal.pone.0288929

**Published:** 2023-07-20

**Authors:** Zhengtao Li, Chen Zhao, Duanhua Li, Lu Wang

**Affiliations:** 1 School of Pharmacy, Qingdao University, Qingdao City, Shandong Province, China; 2 School of Pharmacy, Chengdu University, Chengdu City, Sichuan Province, China; Cukurova University: Cukurova Universitesi, TURKEY

## Abstract

This study presents a multi-factor rational design strategy combined with molecular dynamics simulation to improve the thermostability of *Streptomyces cyaneofuscatus* strain Ms1 tyrosinase. Candidate mutation sites were identified using Discovery Studio and FoldX software, and the double mutant G124W/G137W was obtained. The mutant was heterogeneously expressed in *Escherichia coli* strain Rosetta2 (DE3), and its thermostability was verified. Results indicate that the rational design method, combined with molecular dynamics simulation and protein energy calculation, improved the enzyme’s thermostability more accurately and effectively. The double mutant G124W/G137W had an optimum temperature of 60°C, about 5.0°C higher than that of the wild-type TYRwt, and its activity was 171.06% higher than the wild-type TYRwt. Its thermostability was enhanced, 42.78% higher than the wild-type at 50°C. These findings suggest that the rational design strategy applied in this study can facilitate the application of industrial enzymes in the pharmaceutical industry.

## Introduction

Tyrosinase has a variety of biological functions and can convert L-tyrosine into levodopa (L-DOPA) and melanin. L-DOPA [1] treats Parkinson’s disease, and melanin can kill HIV. Enhancing the thermostability of tyrosinase can aid in the development of more stable and efficient therapeutic interventions, such as enzyme replacement therapies or targeted drug delivery systems. In addition, tyrosinase plays a role in the degradation of pollutants and environmental remediation. It can treat wastewater containing phenol and amines in environmental engineering. Enhancing its thermostability enables the enzyme to function effectively at elevated temperatures, improving its performance in bioremediation processes targeting pollutants that require higher temperatures for degradation. It can be used to catalyze organic synthesis in fine chemicals. Tyrosinase has been used in various electrochemical biosensors to detect phenolic compounds [1–8]. Tyrosinase with enhanced thermostability are highly valuable in biocatalysis, which involves using biological catalysts for chemical reactions. Enhanced thermostability enables the enzyme to withstand higher temperatures, increasing reaction rates and expanding the range of reaction conditions. This can lead to more efficient and sustainable production processes in fields like biotechnology and biofuels. Tyrosine with enhanced thermostability can withstand harsher reaction conditions. This durability enables the use of more sustainable reaction conditions, reducing the need for energy-intensive cooling or purification steps. It can also activate tyrosine residues in peptides to cross-link proteins with chitosan membranes and for direct cross-linking between proteins [9, 10]. The application of tyrosinase in medicine, cosmetology, food and environmental protection has attracted wide attention at home and abroad. Enhancing its thermostability allows the enzyme to maintain its activity and stability at higher temperatures. This enables more efficient and cost-effective production processes by reducing the need for frequent enzyme replacements. Products that contain tyrosinase can benefit from enhanced thermostability. Stability at higher temperatures ensures that the functionality and quality of the products are maintained during storage and usage.

Glycine has the highest conformational entropy among natural amino acids [11], while the smaller and uncharged side chain means it has more rotation space and rotation angle. When the Phi value is greater than zero, glycine can not be changed, and the mutation of glycine to other amino acids will affect the conformation. When the Phi value is less than zero, the glycine mutation into other amino acids with larger side chains helps improve the structural rigidity. This method has been successfully applied to improve the thermostability of many enzymes [12–14].

Alanine scanning mutation (ASM) is often used to analyze the interaction between protein and substrate and to analyze the thermostability of the protein itself. The charged residues on the protein surface are usually not necessary for the protein’s overall structure but generally participate in ligand binding, oligomerization or catalysis. Replacing the charged amino acid residues with alanine residues one by one can eliminate the side chains outside the β-carbon structure and destroy the functional interaction between amino acids without changing the conformation of the main protein chain.

Using molecular dynamics (MD) simulations, conformational changes of proteins can be studied in small time increments (within the range of ps) [15]. The temperature sensitive or conformational flexible regions of proteins can be identified by calculating the root mean square deviation (RMSD) and root mean square fluctuation (RMSF) of the main chain atoms [16].

FoldX [17, 18] is a tool kit for rapid assessment of the effects of mutations on protein stability, folding, and kinetics. The core function of FoldX is to calculate the free energy of macromolecules based on its high-resolution 3D structure. In addition, it can also be used to calculate the stability of proteins, the prediction of proton positions and water bridges, the prediction of metal binding sites, the analysis of free energy of complex formation and alanine scanning. Alibés and Redondo [19, 20] have proved that they can correctly predict energy changes. Discovery Studio is the first software platform that integrates all drug design and biomolecule computing methods and simulation tools under a unified working interface. It is a new generation of molecular modeling and simulation platforms for life science.

In this study, using a rational design strategy, the tyrosinase with higher thermostability was predicted by the combination of molecular dynamics simulation and free energy change, and then the experimental verification also supported this prediction.

## Materials and methods

### Strains, plasmids and reagents

*E*. *coli* strain Top10 (Beijing, China) from TaKaRa and *E*. *coli* strain Rosstta2 (DE3) (Beijing, China) from TaKaRa were used as the host for the gene cloning and protein expression, respectively. The plasmid pACYCDuet-1 and pET28a (Shanghai, China) from Sangon was used as the expression vector. The tyrosinase gene (*TYRwt*) and the metallochaperone gene (*MelC1*) were all from *Streptomyces cyaneofuscatus* strain Ms1 (GenBank accession MF288894) and were synthesized by the General Biotechnology Corporation (Anhui, China). PrimeSTAR Max DNA polymerase and restriction enzyme DpnI was from TaKaRa (Dalian, China). Primers were synthesized by the Sangon Biotechnology Corporation (Shanghai, China). Gel Extraction Kit and Plasmid Mini Kit were purchased from OMEGA Bio-Tek (Guangzhou, China). The genomic DNA extraction, plasmid isolation kits, L-DOPA, chloramphenicol, kanamycin, polyacrylamide and sodium dodecyl sulfate were purchased from Sangon (Shanghai, China). Millipore Amicon^®^Ultra-15 was from Merck (Jiangsu, China). Diethylaminoethyl (DEAE) Bestarose FF anion exchange resin was from Bestchrom (Shanghai, China). All reagents were of analytical grade unless stated otherwise.

### Homology modeling

Since the crystal structure of the tyrosinase from *Streptomyces cyaneofuscatus* strain Ms1 is unknown, Alphafold version 2.1.0 [21] was used for homology modeling of wild-type and mutant tyrosinase. The tyrosinase from *Streptomyces avermitilis* (PDB No. 6J2U) with the best homology replacing Zn^2+^ with Cu^2+^ was used as the template. The models had 73.36% sequence identity with the template. The PROCHECK [22, 23] program is used through the Saves online server, and the Ramachandran Plot [24] is used as a tool to evaluate the molecular conformational rationality of the modeling results.

### Molecular dynamics simulations

The MD simulation of TYRwt was carried out by using NAMD version 2.13 at 300 K and 400 K under the force field of CHARMM27. Created a cube solvent box, placed TYRwt in the center of the box, and set the distance between the enzyme molecule and the edge of the box to be at least 1.5 nm. The minimum cutoff distance between van der Waals and electrostatic interaction was set to 15. Selected the TIP3P water model and add water molecules to the cube box so that the enzyme molecules were solvated entirely in the water box. 9 g/L NaCl was added to the whole solvation system to keep the whole system electrically neutral.

Before MD simulation, energy optimization was needed to avoid the local irrationality existing in the system to affect the simulation results adversely. In this experiment, energy minimization was carried out through four stages: the first stage was to fix the whole protein; the second stage was to remove the restriction of the side chain and only fix the protein skeleton; the third stage was to fix only the secondary structure of the protein to untie the restriction of the loop region; the fourth stage was to release all the restrictions. A 1000-step minimization simulation was carried out for each phase.

The subsequent MD simulation was divided into three steps: first, the constrained MD simulation of 50 ps was carried out in the canonical ensemble (NVT) ensemble, and the system was gradually heated to 300 K; then the constraint was removed, and the MD simulation of 500 ps was carried out in the constant-pressure and constant-temperature (NPT) ensemble to balance the system. Finally, the MD simulation of 20 ns was carried out in the NPT ensemble. It was using the par_all27_prot_na.inp force field parameter, the truncation value (cut off) for non-bond interactions was set to 12.0. Enable Periodic boundary conditions, and Particle mesh enabled to calculate electrostatic interaction. Langevin dynamic simulation and use Langevin piston were used to controlling the system’s pressure at 1 atm, and the time step was set to 2 fs. The VMD version 1.9.3 software package monitored the simulation process, and the conformation was collected every other 50 ps. Calculation and analysis of the average conformation of 5–20 ns in NPT ensemble. The representative structure of the frame conformation closest to the average conformation in the simulation results was selected as the MD simulation results for subsequent analysis.

### Selection of key residues

After the MD simulation, the flexible region of TYRwt was analyzed by comparing the changes in RMSF and RMSD at 300 K and 400 K. The representative structures of 300 K and 400 K molecular dynamics simulation results were compared by Pymol, and the regions with severe deformation were analyzed. The distance between each amino acid residue and the surrounding amino acid residue was measured by VMD. According to the principle of determining whether the Euclidean distance of the β carbon atoms (amino acid residues without β carbon atoms use α carbon atoms) of the two residues was less than three in space, the interaction between the two residues was determined. The deficient contact areas of amino acid residues were analyzed. By calculating the Phi value of Gly, the amino acid residues with a Phi value less than zero were selected for analysis. By using the Calculate Mutation Energy (CME) function of Discovery Studio, the whole enzyme was screened by alanine scanning.

The saturation mutation of the selected sites was carried out by using Discovery Studio to establish the electronic mutation library. The mutation energy was calculated, and the mutation energy <-1.5 kcal/mol was used as the criterion for screening candidate sites. The tyrosinase model was screened, and FoldX was used to evaluate the calculation of energy terms on the platform of YASARA. Based on molecular dynamics analysis and energy analysis, it was found that G124 and G137 had significant effects on the thermostability of TYRwt. Discovery Studio simulated the combined mutation of G124 and G137, and the mutation energy was calculated. We chose the G124W/G137W, which can change the most for experimental verification.

### Site mutagenesis and construction of the recombinant strains

As part of the MelC operon expression product [25–27] tyrosinase from *Streptomyces* is usually required to form heterodimers with caddie protein, with the help of which they fold correctly and acquire enzymatic activity. In an attempt to heterologously express the tyrosinase derived from *Streptomyces* alone, it was found that it could not fold properly and was all expressed as an inclusion body. It is speculated that caddie protein may take up external Cu^2+^ and transfer it to the active center, thus activating tyrosinase activity [28–30]. It has also been suggested that after adding Cu^2+^ into the active center, the tyrosine residues of caddie protein remain dormant in the structural domain of the tyrosinase by acting as competitive inhibitors until the complex dissociates [31–33]. Metallochaperone gene (*MelC1)* and *tyrosinase* genes (*TYRwt*) from *Streptomyces cyaneofuscatus* strain Ms1 were ligated with expression vectors pACYCDuet-1 and pET28a, respectively, to construct recombinant plasmids of pACYCDuet-1-*MelC1* and pET28a-*TYRwt* (S1 Fig). The plasmid carrying the target mutation was obtained by designing primers and using two-point mutations (Table 1). After the end of the PCR reaction, the PCR products were detected by 10 g/L agarose gel electrophoresis, and gel imaging system analyzed the results to determine whether the PCR reaction was successfully amplified. Based on the principle of Dam methylation, the PCR product was digested by the Dpn I enzyme in the water bath at 37°C for 2 h. The recombinant plasmids pACYCDuet-1-*MelC1*, pET28a-*TYR_G124W/G137W*, and pET28a-*TYRwt* were transformed into *E*. *coli* strain Rosetta2 (DE3) competent cells by heat shock, respectively. Single colonies were picked and cultured, plasmids were extracted, and the recombinant mutations were sequenced and verified. Positive transformants were stored at -20°C with a final concentration of 200 g/L glycerol.

**Table 1:** The sequence of primers.

Primer	Template	Sequence(5’-3’)
G124W-F	pET28a-*TYRwt*	GTGAGCTGGGGTCGTTGGCCGGTGACCGTTAC
G124W-R	pET28a-*TYRwt*	CGACCCCAGCTCACTGCAAACGGGCCTTC
G137W-F	pET28a-*TYR_G124W*	GGTCGCTGGTTTCTGCGTCGTGCCCTGGGC
G137W-R	pET28a-*TYR_G124W*	CAGAAACCAGCGACCATCAACGGTAACGGTCACCGGCC

### Heterologous expression of tyrosinase and its variants in *E*. *coli* strain Rosetta2(DE3)

The positive glycerol bacteria were added to the LB medium containing 25 μg/mL chloramphenicol and 50 μg/mL kanamycin with 20 g/L inoculum and cultured overnight at 37°C and 220 rpm. The overnight activated bacterial broth was aspirated at 20 g/L inoculum and added to an LB medium containing 25 μg/mL chloramphenicol and 50 μg/mL kanamycin. After incubation at 37°C and 220 rpm until the absorbance of the bacteria solution reaches 0.6 at 600nm wavelength, β-d-1-thiogalactopyranoside (IPTG) at a final concentration of 0.1 mM was added and incubated for 8 h at 16°C and 220 rpm. Protein expression was analyzed by sodium dodecyl sulfate polyacrylamide gel electrophoresis (SDS-PAGE).

### Enzyme purification

The induced cultures were centrifuged at 8000 rpm for 20 min at 4°C. The precipitate was washed twice with an equal volume of deionized water and suspended in 50 mM Tris-HCl buffer (pH 7.5), disrupted by sonication on an ice water bath, then centrifuged at 10,000 rpm for 30 min at 4°C. The resulting supernatant was added to a solution of saturated ammonium sulfate at a final concentration of 450 g/L, stirred overnight at 25°C, followed by centrifugation at 4°C and 10,000 rpm for 10 min. The precipitate was well suspended in 20 mM sodium phosphate buffer (pH 6.5) buffer and centrifuged at 12000 rpm for 3 min. The resulting supernatant was purified using DEAE Bestarose FF anion exchange resin. Elution of proteins using a linear NaCl gradient (0 to 0.5 M) in the same buffer. Fractions showing tyrosinase activities were pooled, further concentraede with Millipore Amicon^®^Ultra-15 with a molecular weight cut-off of 10 kDa, and stored at 4°C.

### Tyrosinase activity assay

Tyrosinase activity was measured using L-DOPA as a substrate. Oxidation of 10 mM L-DOPA by tyrosinase in 50 μM sodium phosphate buffer (pH 6.5) containing 10 μM CuSO_4_ was monitored using an enzymatic calibrator at 475 nm (ε = 3600 M^-1^ cm^-1^). The amount of enzyme required to increase absorbance by 0.001 per minute at 20±2°C was defined as one unit of enzyme activity [34].

### Thermostability measurements of wild-type and mutant tyrosinase

The optimum temperature was determined by measuring the activity of tyrosinase and mutant enzyme in 50 μM sodium phosphate buffer (pH 6.5) over a temperature range of 20–70°C at 5°C intervals. The highest enzyme activity was defined as 100%, which was used to calculate the relative enzyme activities of the other groups.

Tyrosinase and mutant enzyme were incubated with L-DOPA in 50 μM sodium phosphate buffer (pH 6.5) at temperatures 40,45,50,55 and 60°C. The experiments were performed in triplicate, and the reactions were terminated after 15 min of incubation, and their thermostability was determined by measuring the remaining enzyme activity at 20°C.

### Statistical analysis

Results the average value of the three experimental data was taken, and the error line was taken as the standard deviation, and the significance was analyzed by one-way ANOVA of Graphpadprism9 software (p < 0.05). And use it for data analysis and mapping.

## Results and discussion

### Molecular dynamics simulations to explore the molecular mechanism of thermostability of TYR

**Fig 1:**
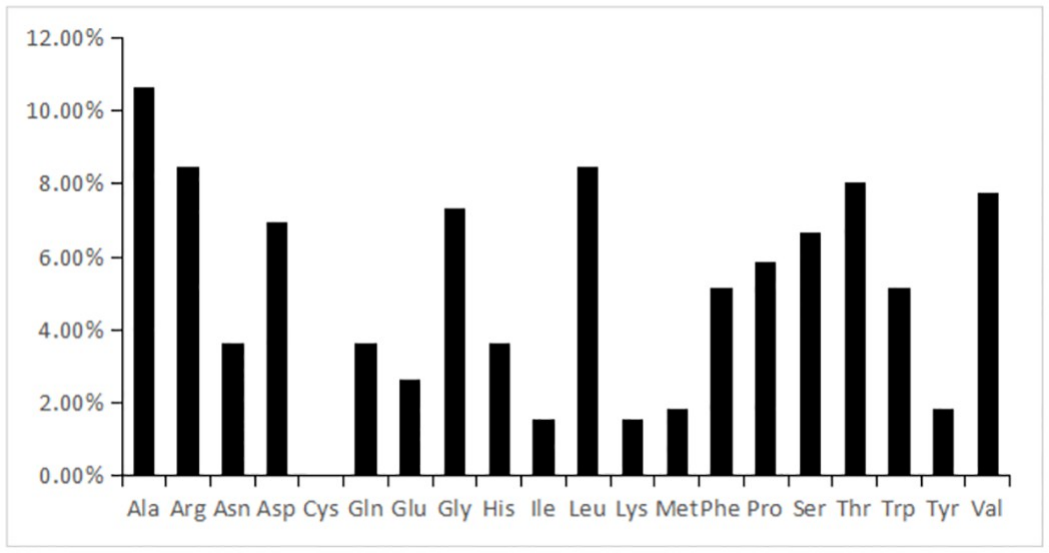
Amino acid composition of tyrosinase.

The analysis of the ProParam online server results revealed that tyrosinase was composed of 274 amino acid residues with a molecular weight of 30.83 kDa. It contained 26 acidic amino acids, 27 basic amino acids, an aliphatic coefficient of 71.24, a total average hydrophobicity of -0.365, and an instability coefficient of 47.84. The overall structure was unstable and had great potential for thermostability modification. According to the ProParam online server analysis, the percentage of each amino acid residue was obtained (Fig 1), and tyrosinase mainly consisted of Ala, Arg, Asp, Gly, Leu, Thr, and Val. At the same time, Ile, Lys, Met, and Tyr were less abundant and did not contain Cys. A large amount of Ala may form α-helix, so it was presumed that the secondary structure of tyrosinase contains a lot of α-helix. Gly’s side chain was smaller and could easily undergo structural changes with temperature changes. A large amount of Gly may be the main reason for the structural instability of tyrosinase.

**Fig 2:**
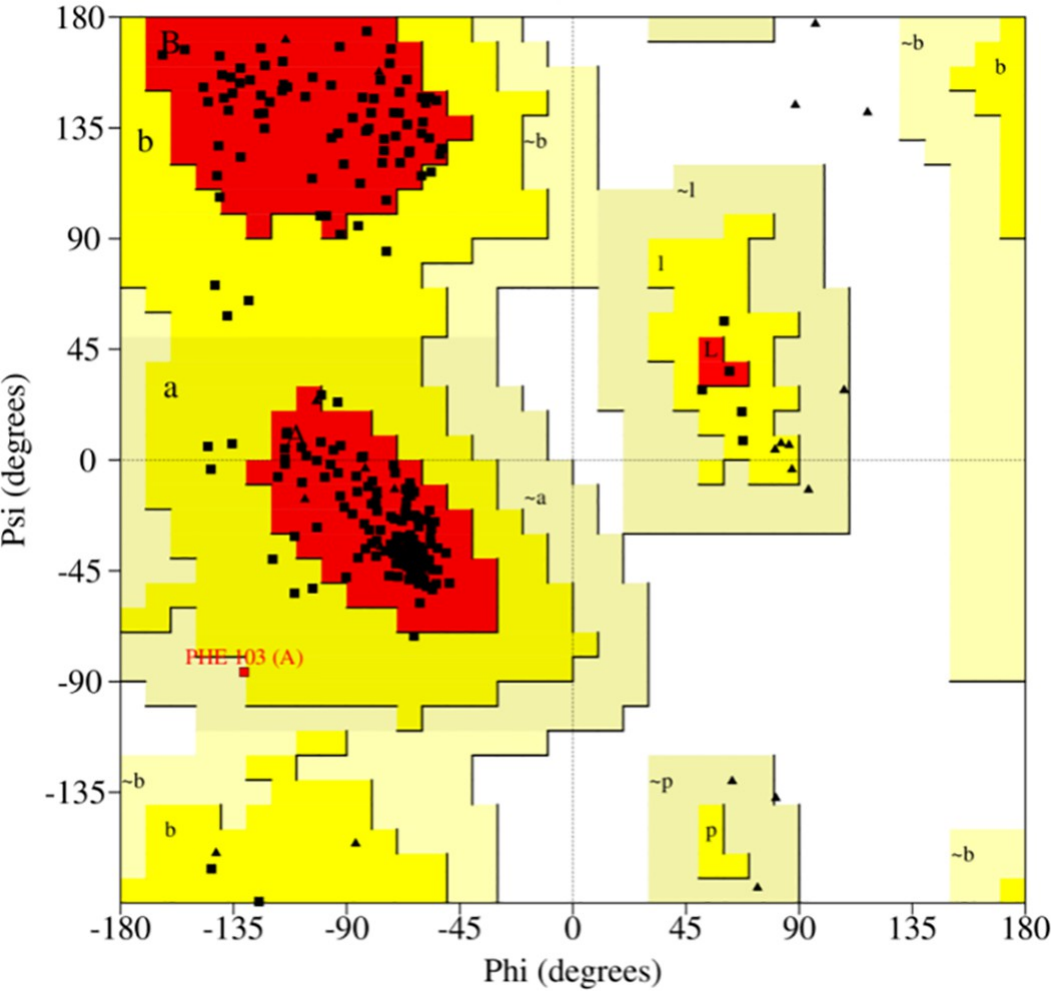
Ramachandran plot.

By amino acid sequence, comparison of the target protein, the tyrosinase with the best homology among them derived from *Streptomyces avermitilis* (PDBid of 6J2U) was selected as a template for homology modeling using Alphafold 2.1.0. The structural model of the final desired tyrosinase was obtained by replacing the two Zn^2+^ in the model with Cu^2+^. The resulting model was evaluated for quality using the Saves server. The Ramachandran plot of the evaluation results showed that all residues in the model were within the reasonable region, with 90.3% of the residues in the optimal region, indicating that the spatial structure obtained from the modeling was reasonable and can be used for subsequent experiments (Fig 2).

**Fig 3:**
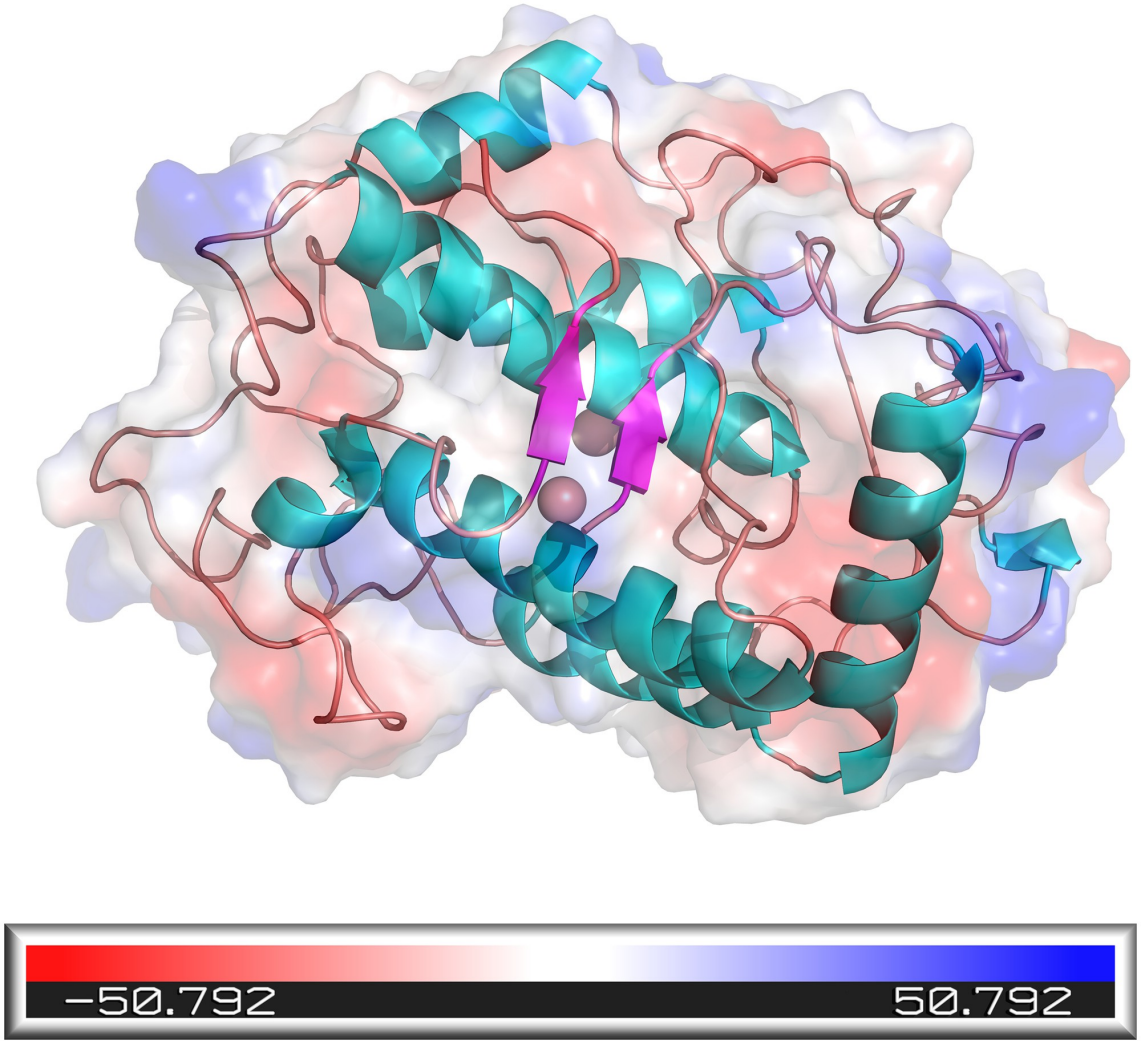
Secondary structure and electrostatic potential energy of TYRwt.

The secondary structure of the established 3D model was analyzed by Pymol and found to be mainly composed of α-helix with a loop. The regions belonging to the random coil were M1, K5-T11, G29, S44-S56, D78-A121, G125-T152, M162-G177, G184-L190, G199-G200, T204-D210, H231-T259, D265-F269 and D273-A276, accounting for up to 59.42% of the total, which was the main secondary structure. The regions belonging to the α-helix structure were A12-T28, Q30-M43, P57-I77, V122-G124, R153-A161, F178-E183, H191-V198, Q201-A203, P211-R230 and P260-L264, which accounts for 38.41% of the total structure. The β-turn regions were T2-R4 and Y269-F272, which accounted for 2.17% of the whole structure (Table 2). The overall structure was alternating random coil with α-helix, consisting of two β-turn at the end of the protein. The α-helix is mainly concentrated in the internal region of the protein and forms the overall rigid framework and active center of the protein (Fig 3). The random coil is mainly located on the protein surface, as well as connecting the individual alpha helices inside. By analyzing the secondary structure of tyrosinase, it was found that the proportion of random coil is higher, more than half of the overall. It means that its structure is more flexible and less stable, and it will change more with increasing temperature, which is an important factor affecting its activity.

**Table 2:** Secondary structure content.

Name	Quantity	Percentage
Alpha helix	106	38.41%
Beta turn	6	2.17%
Random coil	164	59.42%

### Identification and screening of key residue regions

**Fig 4:**
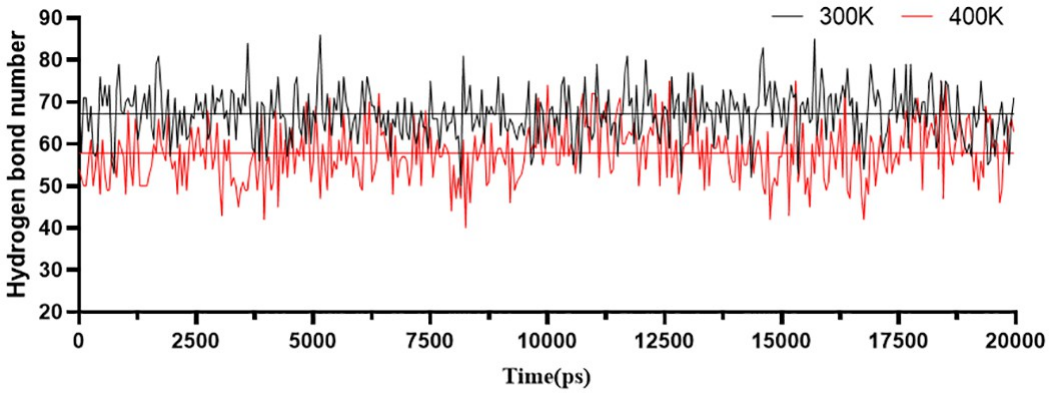
Hydrogen bond quantity diagram.

By recording the changes in the number of hydrogen bonds during the molecular dynamics simulations at 300 K and 400 K, it was found that the overall number of hydrogen bonds fluctuated between 50–86 at 300 K, with an average number of 67. In contrast, the overall number fluctuated between 40–75 at 400 K, averaging 58 (Fig 4). As the temperature increases, the horizontal line in the graph represents a significant decrease in the average number of hydrogen bonds at that temperature. This indicates that the stability of the protein decreases at higher temperatures.

**Fig 5:**
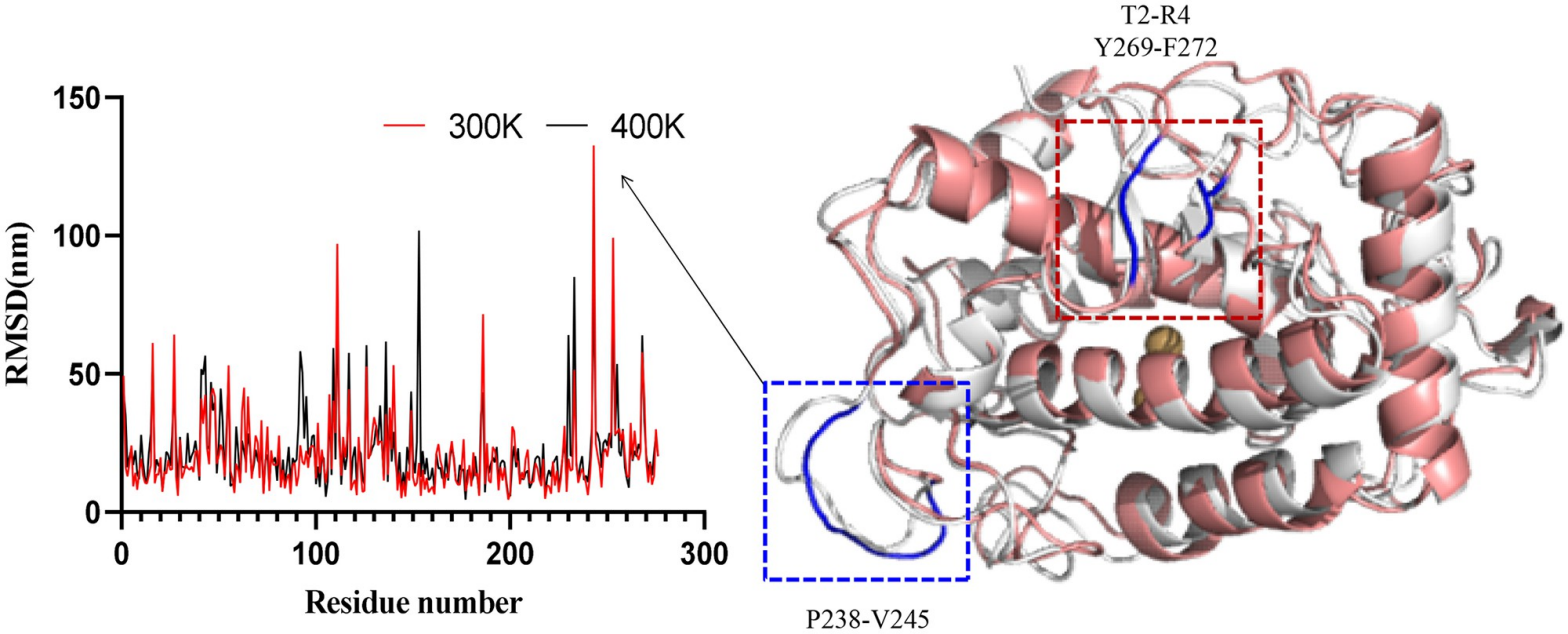
The diagram of RMSD and conformational change of TYRwt at 300 K and 400 K.

By calculating and analyzing the RMSD of each residue during the molecular dynamics simulation at 300 K and 400 K (Fig 5), it was found that there was a significant elevation of RMSD near amino acid residue 240 at 400 K, indicating a significant conformational fluctuation in this region. In cording to the conformation diagrams before and after the temperature rise, it can be seen that the hydrophobic cores of both did not change significantly in conformation during the temperature rise. However, in the loop region of the surface, the structure was significantly looser at 400 K. There was a significant conformational change at the position of amino acid residues 238–245, which corresponds to the RMSD lift near amino acid residue 240 during RMSD analysis. It was found by structural analysis that the N-terminal and C-terminal of the enzyme had a β-fold at 300 K. As the temperature increased, the secondary structure of this region changed at 400 K, and the original β-fold became a loop. Therefore, amino acid residues 2–6 of this region, residues 265–275, and residues 238–245 screened by RMSD changes containing this region, totaling 24 sites, will be used as the region to be modified, and further analysis of the region for modification will follow.

**Fig 6:**
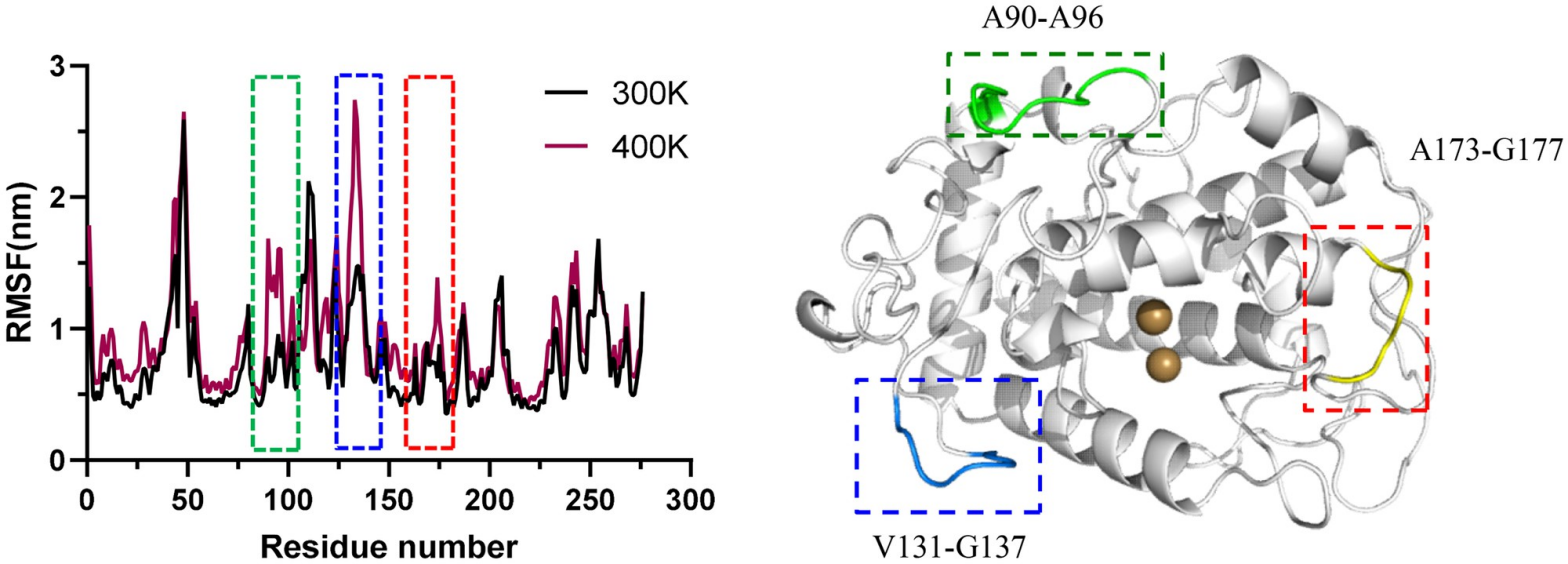
Flexible region analysis. A: RMSF of molecular biology simulations at 300K and 400K; B: Screened transformation areas.

RMSF is an essential parameter in MD simulations that describes the change in the conformation of a protein over time for each amino acid residue relative to the initial structure and is an important indicator of protein flexibility. The regions with considerable flexibility in the spatial protein structure are more prone to conformational changes as the temperature increases. By calculating the RMSF values of the model at 300 K and 400 K, it was found that the RMSF values increased significantly with the increase of temperature in three regions, namely the loop region where residues 90–96 are located, the loop region where residues 131–137 are located, and the loop region where residues 173–177 are located (Fig 6). Combined with the conformational comparison of molecular dynamics simulations, all three regions were located on the protein’s surface, away from the active center. As the temperature increased, the flexibility of this region increased accordingly, and the structure becomes more loose. It is presumed that the region should be an important location affecting the protein’s thermostability. Therefore, these 19 amino acid residues were added to the site to be selected, pending further analysis later.

**Fig 7:**
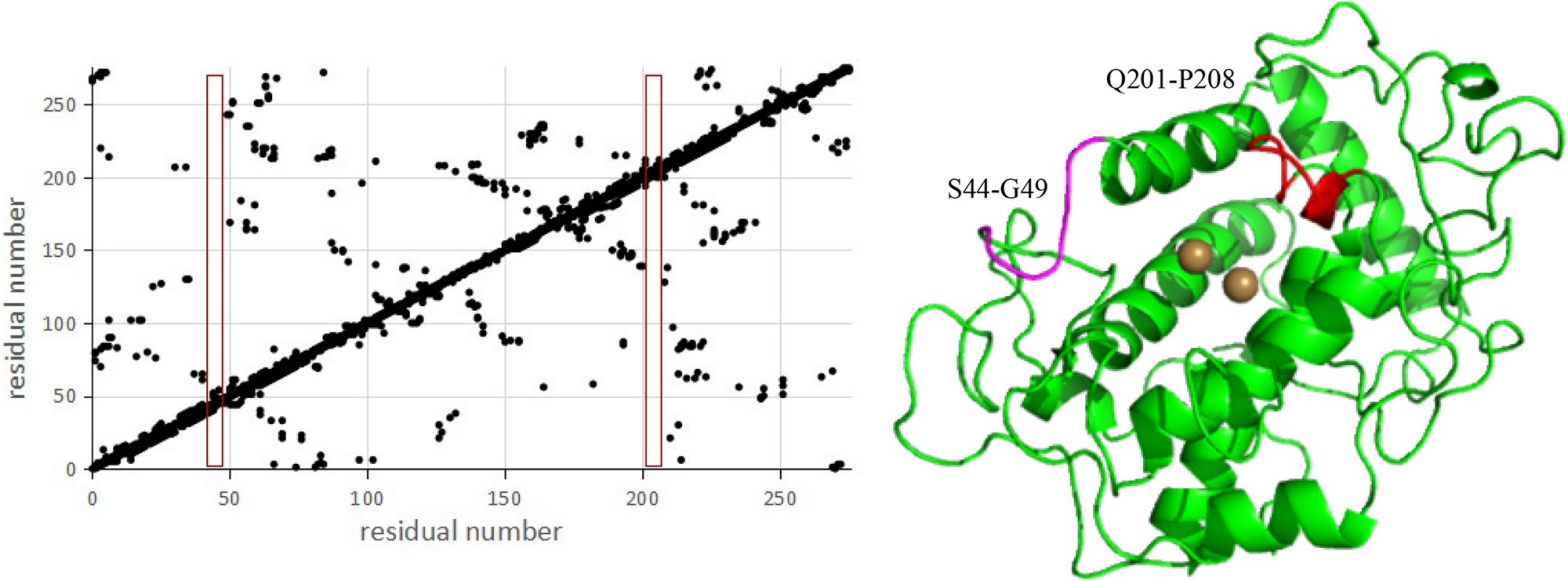
Residue contact analysis. A: Contact of amino acid residues; B: Screened transformation areas.

The interaction between amino acid residues in proteins is an essential factor in determining protein’s spatial structure and stability. By analyzing the residue contacts of each amino acid, the regions with weak residue contacts have less contact with the surrounding amino acid residues. They are, accordingly, more affected by temperature [35]. As the temperature increases, conformational changes are more likely to occur. The analysis of the residue contact map showed that the tyrosinase was in closer contact. Only at residues 44–49 and 201–208 was their less contact with other residues (Fig 7). Residues 201–208 were in the substrate pocket beside the active site. The modification of this region may cause significant changes in enzyme activity. It may even lead to inactivity of the enzyme, so it was not considered as a modification region. Residues 44–49 were located on the protein’s surface, away from the active site. Their modification had less effect on the enzyme activity, so their six sites were added to the sites to be selected and await further analysis.

**Fig 8:**
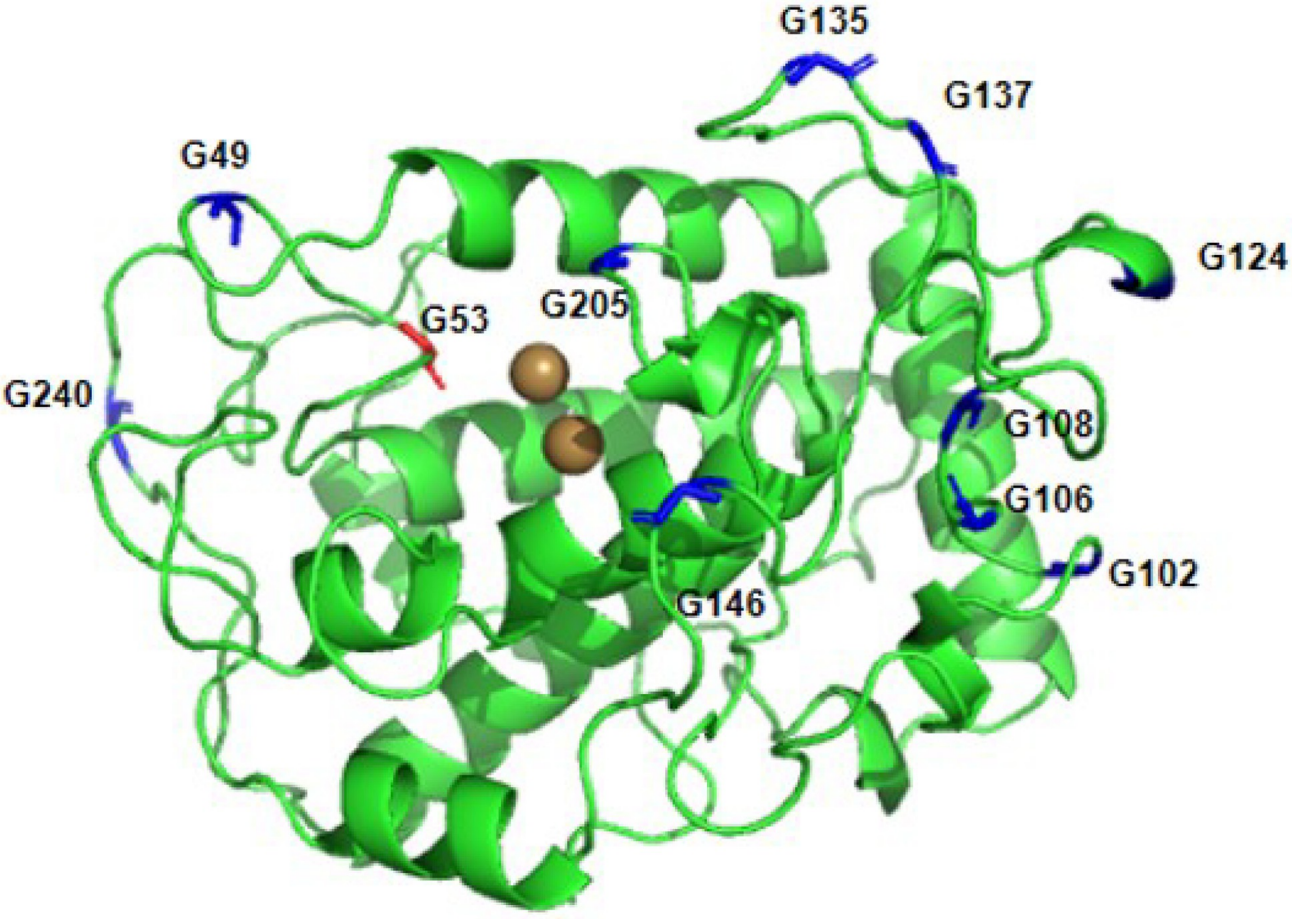
Glycine sites screened from loop region.

All 22 glycine residues in the protein molecule were calculated, and 11 glycine sites with Phi values lower than zero were identified (Table 3). One of them, G53, was located directly besides the active site. To avoid any effect on the enzyme’s catalytic activity, we excluded site 53. The remaining G49, G102, G106, G108, G124, G135, G137, G146, G205 and G240, a total of 10 amino acid residues, were added to the site to be selected pending further screening (Fig 8).

**Table 3:** Phi value of glycine.

Residue	Phi
29	77.5
49	-126.8
53	-119.7
102	-87.6
105	98.5
106	-57.8
108	-36.9
113	83.8
118	109
124	-78.8
125	77.2
135	-83.7
137	-66.4
144	81.2
146	-84.4
177	81.7
184	75.1
187	57.2
199	44.1
200	49.9
205	-73.3
240	-87.4

**Fig 9:**
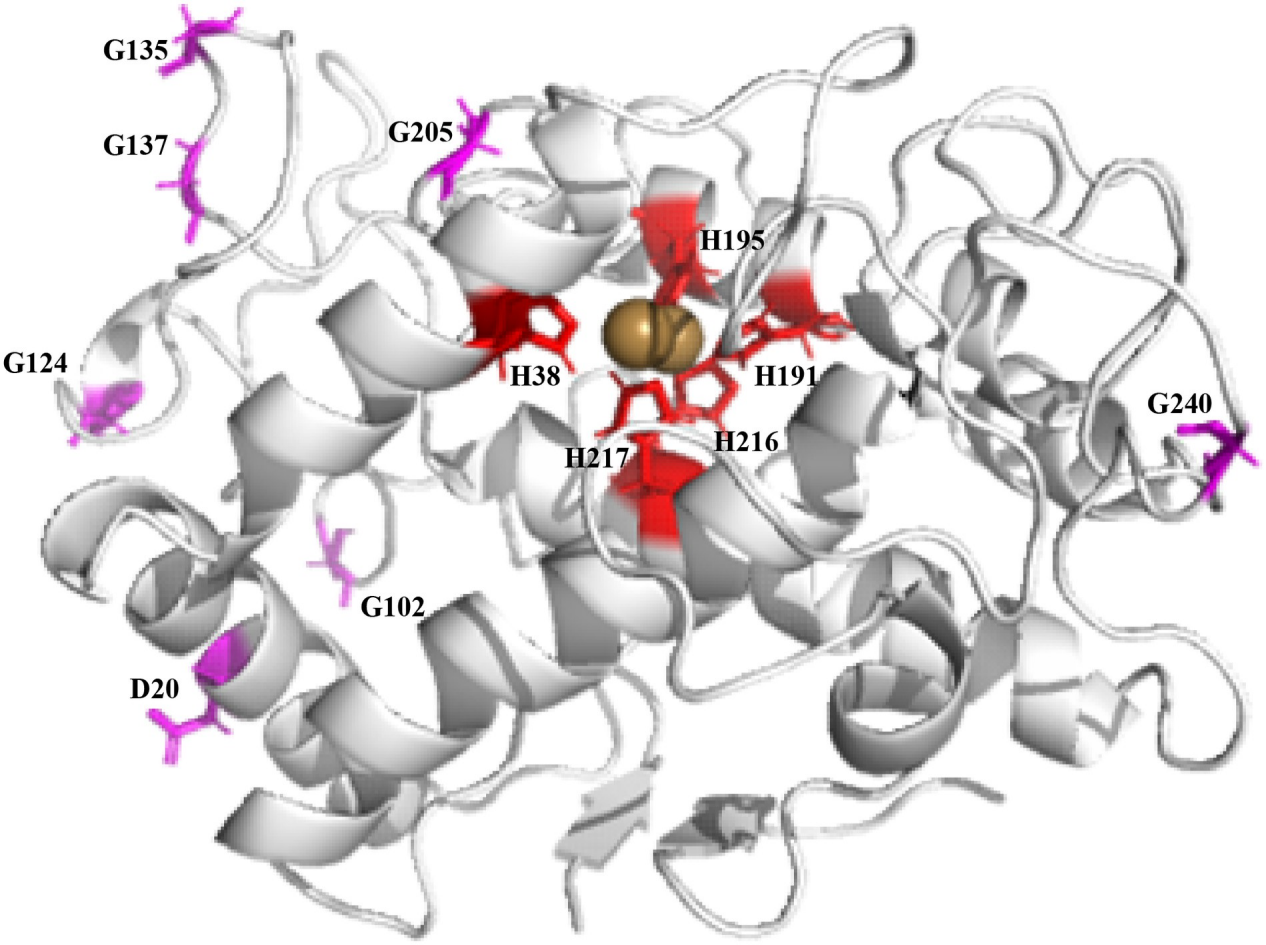
Selected modification site.

All amino acid residues were mutated to alanine one by one by using Discovery Studio software, and the mutation energy was calculated. If the mutation energy is between -0.5 kcal/mol and +0.5 kcal/mol, this mutation dose not effect stability. The mutation energy is above 0.5 kcal/mol, and it is considered that this mutation may lead to a decrease in stability. Otherwise, if the mutation energy is below -0.5 kcal/mol, this mutation may increase stability. A total of 12 sites with mutation energies below -0.5 kcal/mol were obtained (Table 4), among which H38, H191, H195, H216, and H217 are active sites for binding to copper ions, and mutating these sites to other amino acid residues would result in a substantial decrease in activity (Fig 9). Therefore, seven remaining amino acid residues, D20, G102, G124, G135, G137, G205, and G240, were added to the sites to be selected pending further screening.

**Table 4:** Mutation energy of alanine scanning mutation.

Residue	Mutation Energy(kcal/mol)
217	-2.52
191	-2.06
195	-2.01
124	-1.54
216	-1.33
137	-1.01
20	-0.84
38	-0.84
205	-0.81
102	-0.62
135	-0.56
240	-0.56

### Identification of mutants based on mutation energy

All the above screened 56 sites were aggregated, and the saturation mutations were performed using each of the screened site, and their mutation energies were calculated. Among them, the mutation energy of G124W and G137W was -3.82 kcal/mol and -3.58 kcal/mol, respectively, far lower than other mutants (Table 5). The mutation energies of all 19 mutations in residue 137 were lower than zero, and only one mutation in residue 124 was above zero (Table 6). This indicates that these two loci may be essential sites affecting protein stability, and the mutants exhibited are inclined to higher stability. Double site saturation mutations were performed on G124 and G137 sites. G124W/G137W possessed the lowest mutation energy, so G124W/G137W was subjected to experimental validation.

**Table 5:** Mutation energy of saturation mutants.

Mutation	G124 Mutation Energy(kcal/mol)	G137 Mutation Energy(kcal/mol)
ALA	-1.54	-1.01
ARG	-2.59	-1.28
ASN	-3.12	-1.46
ASP	-1.24	-0.72
CYS	-2.49	-2.12
GLN	-2.05	-0.8
GLU	-2.67	-1.24
GLY	0	0
HIS	-2.67	-1.05
ILE	-2.36	-2.25
LEU	-3.74	-2.15
LYS	-0.21	-1.21
MET	-2.26	-2.07
PHE	-3.14	-2.83
PRO	5.28	-1.66
SER	-1.51	-1.32
THR	-1.74	-1.75
TRP	-3.82	-3.58
TYR	-3.03	-2.84

**Table 6:** Mutation energy of double saturated mutants.

Mutation	G124/G137 Mutation Energy(kcal/mol)
G124W/G137W	-7.42
G124L/G137W	-7.26
G124L/G137Y	-6.76
G124W/G137F	-6.71
G124W/G137Y	-6.7
G124N/G137W	-6.67
G124L/G137I	-6.65
G124Y/G137W	-6.54
G124F/G137F	-6.47
G124L/G137F	-6.46
G124F/G137W	-6.39
G124H/G137W	-6.34
G124W/G137I	-6.24
G124F/G137Y	-6.21
G124L/G137Q	-6.16

### Construction, expression and purification of recombinant enzymes

**Fig 10:**
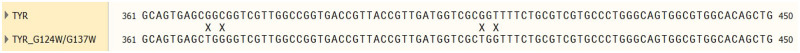
Sequence comparison diagram.

A recombinant plasmid containing the target mutation was obtained by two point mutations using pET28a-*TYRwt* as a template. The obtained PCR products were verified by electrophoresis and showed clear bands between 5000bp and 7500bp (S2 Fig), consistent with the theoretical value of molecular weight 6060bp. After verification by plasmid sequencing, the resulting DNA fragment was determined to be consistent with the sequence of the target plasmid, and the mutation pET28a-*TYR_G124W/G137W* containing the target mutation was successfully obtained (Fig 10).

pACYCDuet-1-*MelC1* was transformed into the expression host *E*. *coli* strain Rosetta2(DE3) competent cell simultaneously with pET28a-*TYRwt* and pET28a-*TYR_G124W/G137W*, respectively, to achieve dual protein expression. Under the resistance pressure of chloramphenicol and kanamycin, several single colonies were formed in the solid medium (S3 Fig). The plasmids were extracted from two single colonies. After agarose gel electrophoresis, there were obvious bands around 3997 bp and 6133 bp, consistent with the theoretical molecular weight of 4348 bp of pACYCDuet-1-*MelC1* and 6060 bp of pET28a-*TYRwt* and pET28a-*TYR_G124W/G137W* (S4 Fig). The results showed that the two recombinant plasmids were successfully transformed into host cells at the same time. The two bands above should be unrepaired ring-opening plasmids.

**Fig 11:**
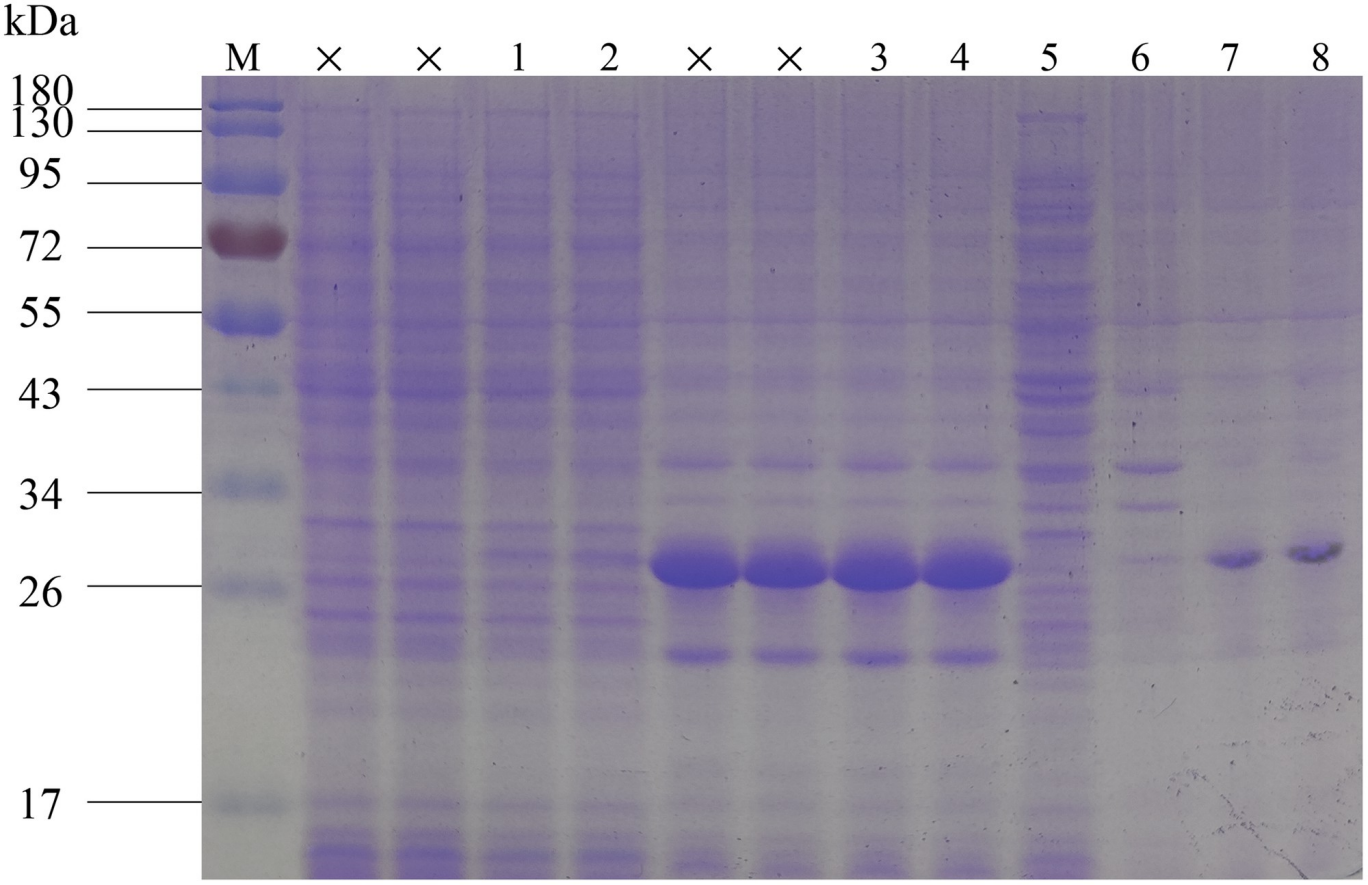
SDS-PAGE of expression product. M: PageRuler Prestained Protein Ladder; 1: TYRwt supernatant with added inducer; 2: G124W/G137W supernatant with added inducer; 3: TYRwt precipitate with added inducer; 4: G124W/G137W precipitate with added inducer; 5: G124W/G137W supernatant without added inducer; 6: G124W/G137W precipitate without added inducer; 7: Purified TYRwt; 8: Purified G124W/G137W.

The constructed engineering bacteria were induced to express, and SDS-PAGE analysis showed band formation in 14.5 kDa, which proved that caddie protein MelC1 was expressed successfully (Fig 11). The soluble expression products of TYRwt and G124W/G137W have obvious bands between 26 kDa band and 34 kDa band, consistent with the target protein’s molecular weight of 30 kDa. After the supernatant enzyme was purified by anion chromatography, the target protein was purified as the main band.

### Study on temperature and thermostability

**Fig 12:**
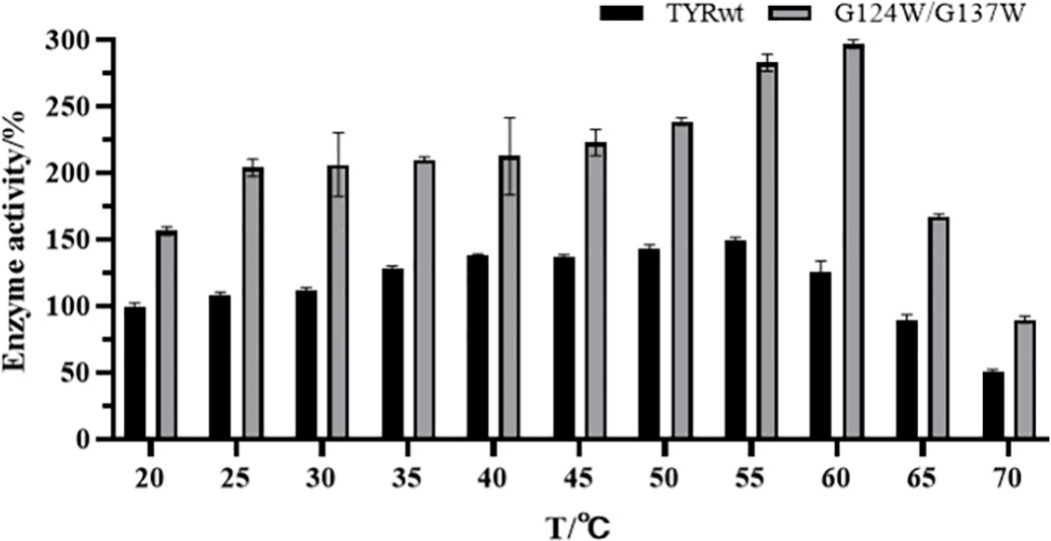
The optimum temperature of TYRwt and G124W/G137W.

Using L-DOPA as substrate, the activity of the double mutant and the wild strain was measured at different temperatures. It was found that double mutant G124W/G137W showed higher activity at high temperatures (Fig 12). Among them, the optimum temperature of mutant G124W/G137W was 60°C, which was five centigrade higher than that of wild tyrosinase. With the enzyme activity defined as 100% at 20°C, the double mutant G124W/G137W activity at 60°C was 296.64%, much higher than the maximum activity of the wild-type tyrosinase at 149.82%.

**Fig 13:**
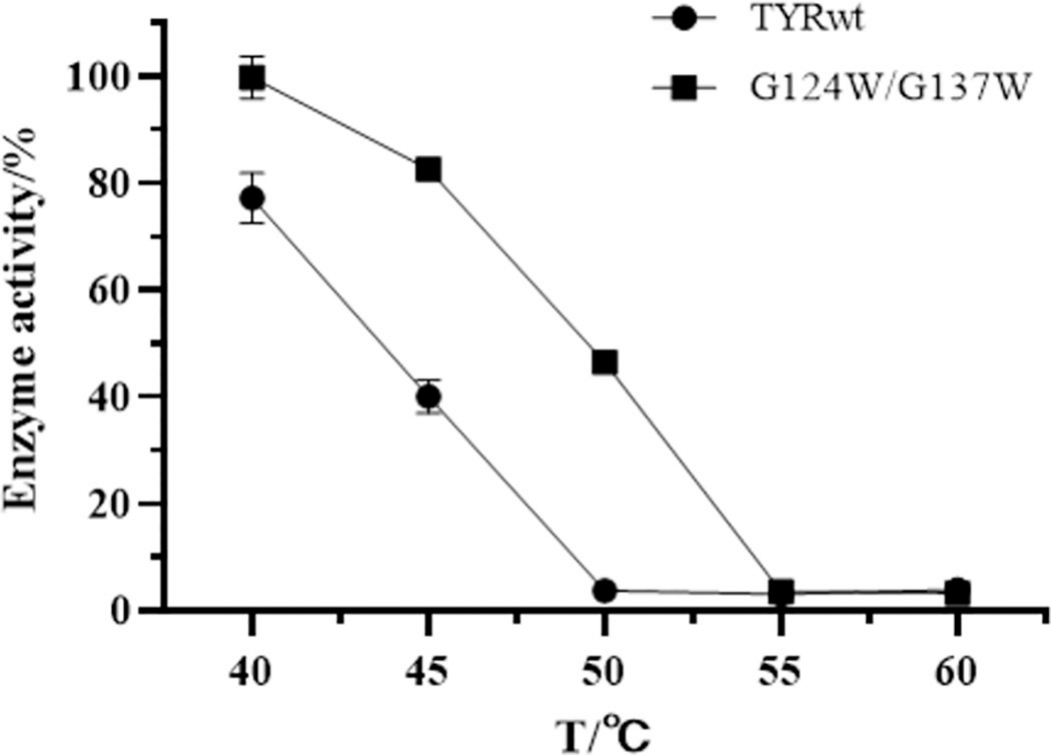
Thermostability of TYRwt and G124W/G137W.

The residual activity after holding treatment at 40–60°C for 15 min was used to evaluate the thermostability of the wild-type with the double mutant G124W/G137W. The enzyme activity without heat treatment was defined as 100%, which was used to calculate the relative enzyme activity of the other groups. TYRwt showed good thermostability at 40°C (Fig 13). With increasing temperature, its thermostability decreases rapidly, losing 60% of its activity at 45°C. It was almost wholly inactivated within 15 min at 50°C. In contrast, the thermostability of mutant G124W/G137W was significantly improved, retaining more than 80% activity at 45°C for 15 min of treatment. It retained 45% activity at 50°C for 15 min of activity treatment, which was 42.78% higher than the wild-type. It was almost inactivated at 55°C. The above results suggest that the G124W/G137W double mutant improves the thermostability of tyrosinase.

### Mutants for energy analysis

Using Yasara as a platform, the Positionscan program in the FoldX plugin can saturate and mutate each amino acid residue into nineteen other amino acid residues and derive their folding free energy change (ΔΔG). A negative value of ΔΔG indicates a decrease in energy after mutation, and the larger the absolute value, the more stable the conformation after mutation. It was calculated that ΔΔG for mutation of glycine residue 124 to tryptophan is -0.996959 kcal/mol and ΔΔG for mutation of glycine residue 137 to tryptophan is -0.984701 kcal/mol in the wild-type enzyme. Therefore, the ΔΔG of the double mutant enzyme is -1.98166 kcal/mol. The significant decrease of ΔΔG indicates that the overall folding free energy becomes smaller after the mutation, resulting in a better stability under high temperature conditions.

Compared with other modeling software, AlphaFold 2.1.0 uses a method based on deep learning, especially the deep convolution neural network, which enables it to learn complex patterns and dependencies in protein structures. This architecture allows for better capture of remote interactions and overall structural characteristics, ensuring a comprehensive understanding of protein folding patterns and interactions, which leads to more accurate predictions in this experiment. AlphaFold2.1.0 provides confidence score for the prediction of tyrosinase structure. It makes it possible to evaluate the reliability of the prediction model and facilitate downstream analysis and decision-making.

Based on the md results, the six characteristics of the flexible zone are analyzed, and the candidate regions are selected by combining the results, and then the re-screening method of folding free energy is carried out. This method not only saves the time-consuming virtual screening process based on molecular docking, but also can obtain effective results. In the future, this process can be used to design and modify the stability of different types of enzymes, in order to further prove the effectiveness of this method.

## Conclusion

This study proposed a computational design scheme based on the combination of molecular dynamics simulation and energy calculation to improve tyrosinase’s thermostability. Firstly, molecular dynamics simulations are carried out on the TYRwt molecular model at 300 K and 400 K, respectively. According to the conformational change of MD at high temperature, RMSF calculation, contact analysis and Phi value analysis of Gly, 56 unstable regions were screened from the 276 amino acid residues of tyrosinase. Then two key mutation sites G124 and G137 were obtained by using mutation energy calculation and ΔΔG calculation. Finally, the double mutant G124W/G137W was screened by simulating combinatorial mutation, combined with molecular dynamics analysis and energy change, and verified by experiments. It was found that the optimum temperature of the double mutant G124W/G137W was 60°C, which was about five centigrade higher than that of the wild-type TYRwt. Its catalytic activity is 171.06% higher than wild-type TYRwt at 60°C. Moreover, its thermostability was enhanced, 42.78% higher than the wild-type under 50°C. The results show that the combination of molecular dynamics simulation and energy calculation will be an accurate, rational strategy to improve the thermostability of the enzyme.

## Supporting information

S1 Fig Construction of recombinant plasmid pACYCDuet-1-*MelC1* and pET28a-*TYRwt*. (TIF)

S2 Fig PCR verification diagram of pET28a-*TYR_G102W/G205Y* and pET28a-*TYR_G124W/G137W*. M: DNA Marker IV; 1: pET28a-*TYR_G124W/G137W*.
(TIF)

S3 Fig The map of transformant. (TIF)

S4 Fig The map of plasmid verification. 1: pACYCDuet-1-*MelC1*/pET28a-*TYRwt*; 2: pACYCDuet-1-*MelC1*/pET28a-*TYR_G124W/G137W*; M: Supercoiled DNA Ladder Marker.
(TIF)

S1 Raw images (PDF)
