# Supplementary figures and images for: Enhancing the thermostability of Streptomyces cyaneofuscatus strain Ms1 tyrosinase by multi-factors rational design and molecular dynamics simulations
Source: PLoS One. 2023 Jul 20;18(7):e0288929. doi: 10.1371/journal.pone.0288929 (PMC10358999; doi:10.1371/journal.pone.0288929)

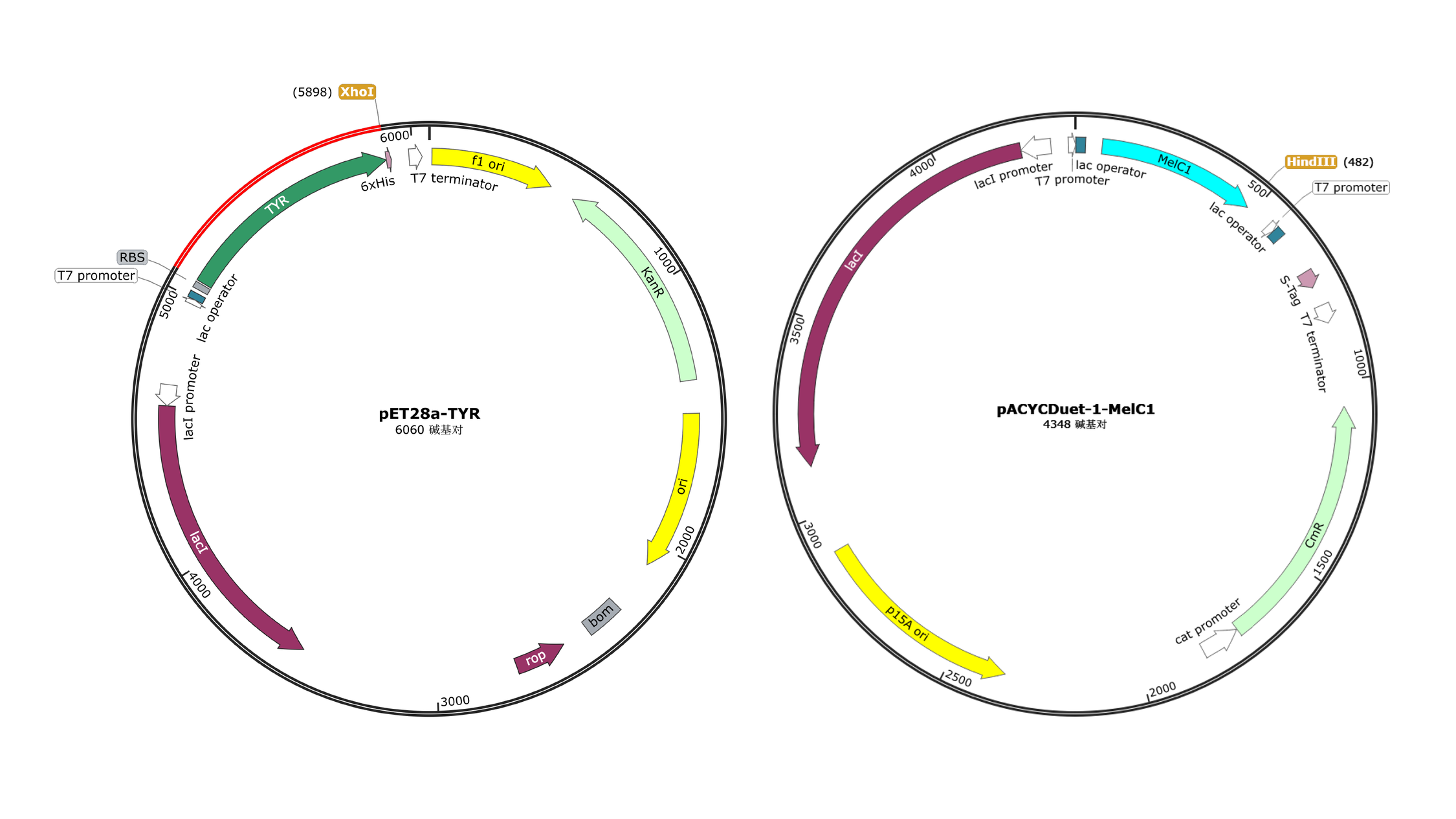

Supplement: S1 Fig — (TIF) [file pone.0288929.s001.tif]

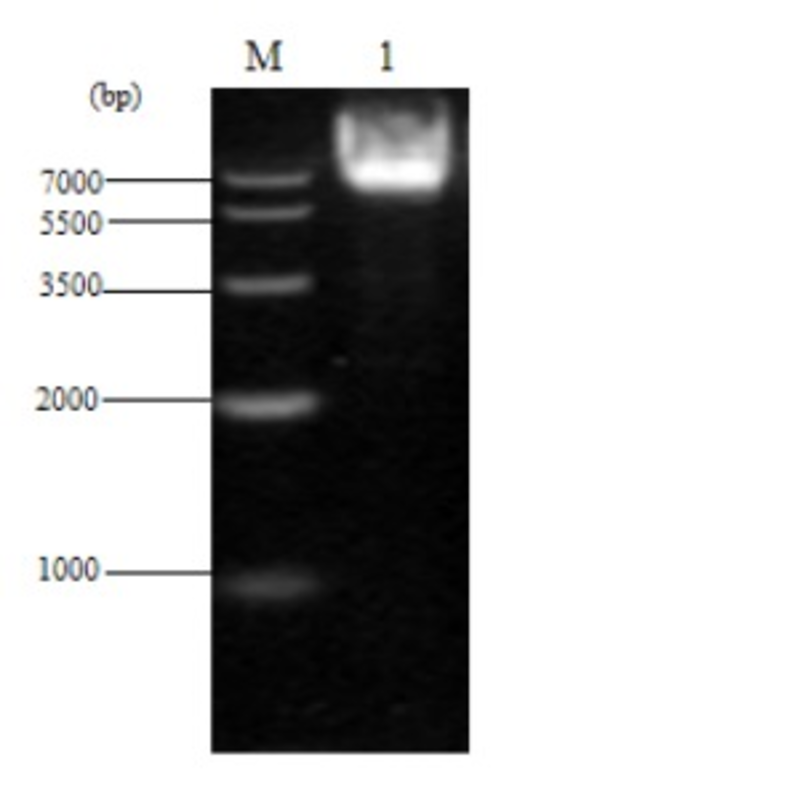

Supplement: S2 Fig — M: DNA Marker IV; 1: pET28a-TYR_G124W/G137W. (TIF) [file pone.0288929.s002.tif]

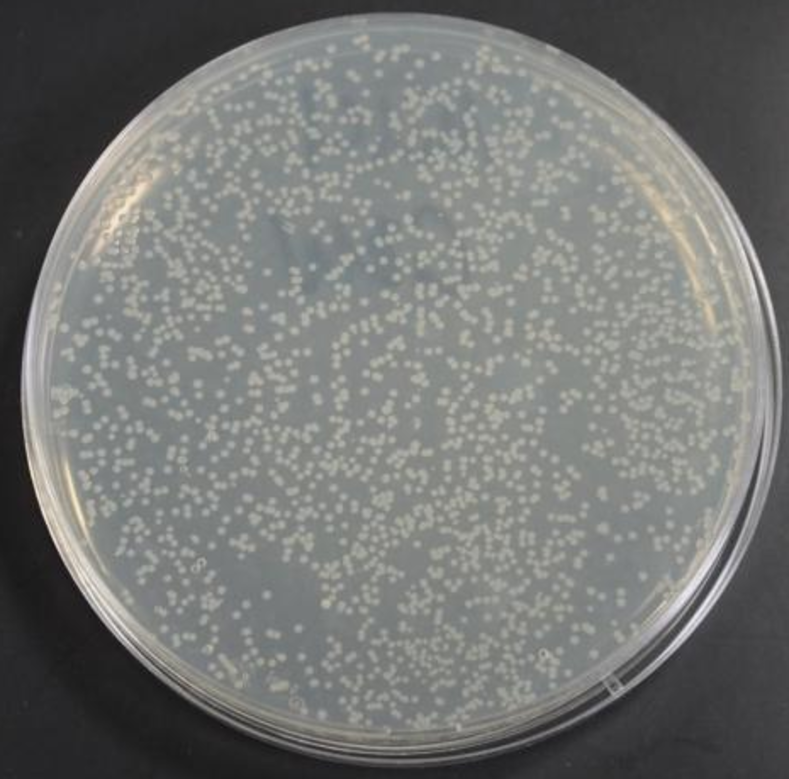

Supplement: S3 Fig — (TIF) [file pone.0288929.s003.tif]

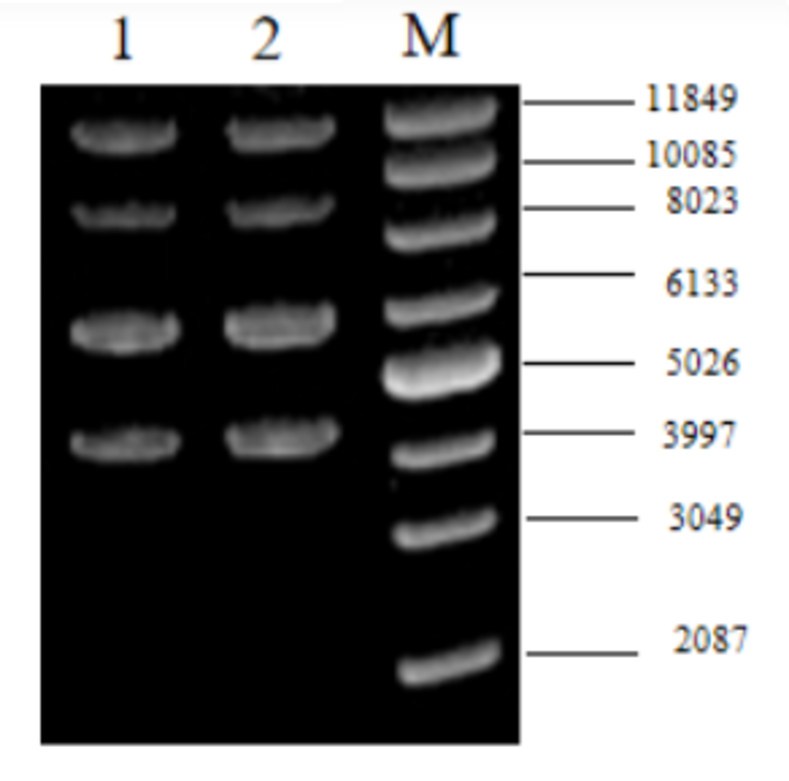

Supplement: S4 Fig — 1: pACYCDuet-1-MelC1/pET28a-TYRwt; 2: pACYCDuet-1-MelC1/pET28a-TYR_G124W/G137W; M: Supercoiled DNA Ladder Marker. (TIF) [file pone.0288929.s004.tif]
