# Supplementary material for: Enhancing the thermostability of Streptomyces cyaneofuscatus strain Ms1 tyrosinase by multi-factors rational design and molecular dynamics simulations
Source: PLoS One. 2023 Jul 20;18(7):e0288929. doi: 10.1371/journal.pone.0288929 (PMC10358999; doi:10.1371/journal.pone.0288929)
Supplement: S1 Raw images — (PDF) [file pone.0288929.s005.pdf]

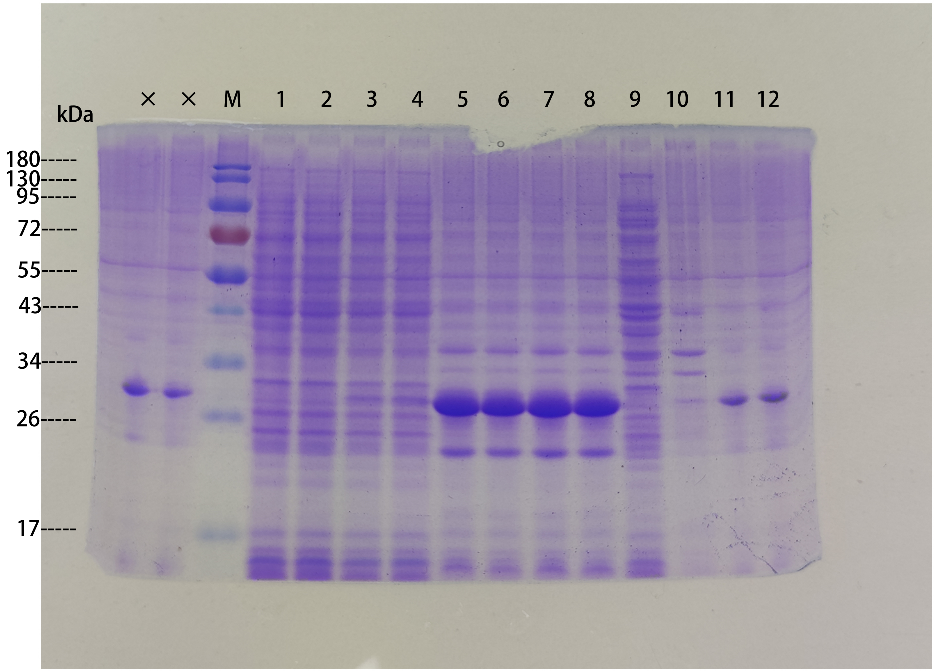

M: PageRuler Prestained Protein Ladder;

1: G49F/G205W supernatant with added inducer;

2: G102W/G205Y supernatant with added inducer;

3: G124W/G137W supernatant with added inducer;

4: TYRwt supernatant with added inducer;

5: G49F/G205W precipitate with added inducer;

6: G102W/G205Y precipitate with added inducer;

7: G124W/G137W precipitate with added inducer;

8: TYRwt precipitate with added inducer;

9: G124W/G137W supernatant without added inducer;

10: G124W/G137W precipitate without added inducer;

11: Purified TYRwt;

12: Purified G124W/G137W

The gel imaging system is ChemiDoc XRS from Biorad.

The order of samples is from left to right.
